# The Anti-Adipogenic Potential of COUP-TFII Is Mediated by Downregulation of the Notch Target Gene Hey1

**DOI:** 10.1371/journal.pone.0145608

**Published:** 2015-12-31

**Authors:** Ilse Scroyen, Dries Bauters, Christine Vranckx, H. Roger Lijnen

**Affiliations:** KU Leuven, University of Leuven, Department of Cardiovascular Sciences, Center for Molecular and Vascular Biology, B-3000, Leuven, Belgium; Tohoku University, JAPAN

## Abstract

**Background:**

Chicken ovalbumin upstream promoter transcription factor II (COUP-TFII) belongs to the steroid/thyroid hormone receptor superfamily and may contribute to the pathogenesis of obesity. It has not conclusively been established, however, whether its role is pro- or anti-adipogenic.

**Methods and Results:**

Gene silencing of *Coup-tfII* in 3T3-F442A preadipocytes resulted in enhanced differentiation into mature adipocytes. This was associated with upregulation of the Notch signaling target gene *Hey1*. A functional role of Hey1 was confirmed by gene silencing in 3T3-F442A preadipocytes, resulting in impaired differentiation. *In vivo*, *de novo* fat pad formation in NUDE mice was significantly stimulated following injection of preadipocytes with *Coup-tfII* gene silencing, but impaired with *Hey1* gene silencing. Moreover, expression of *Coup-tfII* was lower and that of *Hey1* higher in isolated adipocytes of obese as compared to lean adipose tissue.

**Conclusions:**

These *in vitro* and *in vivo* data support an anti-adipogenic role of COUP-TFII via downregulating the Notch signaling target gene *Hey1*.

## Introduction

Over the last decades obesity and its consequences worldwide have become a major health problem. Indeed, obesity is associated with an increased mortality from cardiovascular disease, some forms of cancer, diabetes and kidney disease [1].

Chicken ovalbumin upstream promoter-transcription factor II (COUP-TFII, also known as NR2F2, nuclear receptor subfamily 2 group F, member 2) is a nuclear orphan receptor that belongs to the steroid/thyroid hormone superfamily and may contribute to the pathogenesis of obesity. COUP-TFII was reported to play an important role in adipogenesis and energy homeostasis [2], and it has a documented role in tumor angiogenesis through the regulation of Ang1/Tie2 signaling or VEGF/VEGF receptor 2 signaling [3]. *Coup-tfII* is expressed in adipocytes as well as in the vascular compartment of mainly white adipose tissue, but recent reports on its role in adipogenesis have been contradictory [2, 4, 5]. *Coup-tfII* expression decreases during differentiation of preadipocytes and its overexpression impairs adipogenesis by repressing the expression of pro-adipogenic factors in adipocytes [5]. In addition, reduction of *Coup-tfII* mRNA expression allows fibroblasts to differentiate into fat cells, indicating that COUP-TFII acts downstream of the hedgehog signaling and is required for the full anti-adipogenic effect of this pathway [5]. In agreement, Okamura et al. reported that Wnt/beta-catenin signaling activates the expression of *Coup-tfII*, which in turn represses *PPAR*γ gene expression, resulting in inhibition of adipogenesis [4]. In contrast, reduced adipose tissue mass and improved glucose homeostasis were shown in heterozygous COUP-TFII mice as compared to wild-type (WT) mice [2]. It is thus not conclusively established whether COUP-TFII has a pro- or anti-adipogenic potential.

COUP-TFII is considered to be a major regulator of Notch signaling pathways [6]. COUP-TFII homodimers inhibit arterial differentiation of venous endothelial cells through direct binding to the promoter regions of the Notch target genes H*ey1* and H*ey2*, causing transcriptional repression [7]. Interestingly, it was shown that endothelial cells and adipocytes have a common progenitor [8]. Conflicting data have been reported on the role of Notch signaling in adipogenesis. Garces et al. first showed that Notch1 is required for adipogenesis [9], whereas later it was argued that Notch is dispensable in adipocyte specification [10]. More recently it was reported that inhibition of canonical Notch signaling inhibited adipogenesis [11], whereas activation of this pathway stimulated adipogenesis [12]. In conflict with these data, it was reported that inhibition of Notch signaling promotes differentiation of preadipocytes [13–15]. In addition, a dual role for the Notch target gene *Hes1* in adipocyte development was suggested [15], whereas the role of H*ey1* in adipogenesis was not further explored.

To clarify the functional role of COUP-TFII and Notch-Hey1 signaling in adipogenesis, we used established mouse models of *in vitro* adipocyte differentiation and *in vivo* adipogenesis.

## Materials and Methods

### In vitro models

#### Gene silencing in 3T3-F442A preadipocytes

To obtain long term stable gene silencing of *Coup-tfII* or H*ey1* in 3T3-F442A preadipocytes [16], the ‘MISSION shRNA lentiviral transduction particles’ system (Sigma-Aldrich, St. Louis, MO) was used as described elsewhere [17]. For COUP-TFII (NM_009697), five different clones were tested (TRCN0000026167-026232-054474-054475-312204), as well as for the Notch signaling target gene Hey1 (NM_010423; clones TRCN0000086479-86480-86481-86482 and TRCN0000311840). MISSION non-target shRNA control transduction particles (SHC002V) were used as negative control. Puromycin-resistant preadipocytes with or without *Coup-tfII* or *Hey1* gene silencing were differentiated into mature adipocytes as described below.

#### Culture and differentiation of 3T3-F442A preadipocytes

3T3-F442A murine preadipocytes were grown in DMEM (ThermoFisher Scientific, Gent, Belgium) supplemented with 10% fetal bovine serum (ThermoFisher Scientific) and 5% penicillin/streptomycin (ThermoFisher Scientific) and were passaged when pre-confluent. To induce differentiation, cells were seeded at a density of 25 x 10^3^ cells/cm^2^ and differentiated as described elsewhere [18]. To monitor the extent of differentiation, RNA was collected at different time points, and cultures were stained with Oil Red O, as described [18, 19]. Cell extracts of preadipocytes (day 0) and differentiated cells (day 12) were prepared in RIPA buffer (Sigma-Aldrich) and the protein concentration was measured with the bicinchoninic acid method (ThermoFisher Scientific) according to the manufacturer’s instructions. Samples were stored at -80°C

#### DAPT treatment

To efficiently block the γ-secretase complex, thereby completely blocking Notch responses during differentiation, 3T3-F442A cells were treated with 10 μM of *N-*[*N*-(3,5-Difluorophenacetyl-L-alanyl)]-(*S*)-phenylglycine *t*-butyl-ester (DAPT; Calbiochem, San Diego, CA) dissolved in DMSO.

### In vivo and ex vivo models

#### *De novo* adipogenesis *in vivo*

To induce *de novo* fat pad formation, 10 x 10^6^ 3T3-F442A preadipocytes (with or without gene silencing), grown to near confluency and resuspended in phosphate buffered saline (PBS), were injected subcutaneously in the back of 6 week old male athymic Balb/c NUDE mice (Charles River, Les Oncins, France) [20–22]. Mice were kept in microisolation cages on a 12h day/night cycle and fed with high fat diet (HFD, Harlan Teklad TD88137, Zeist, The Netherlands; 42% kcal as fat, caloric value 20.1 kJ/g) for 4 weeks. Body weight was measured weekly. At the end of the experiment, after 6 hours fasting, mice were anesthetized by intraperitoneal injection of 60 mg/kg Nembutal (Abbott Laboratories, North Chicago, IL). Intra-abdominal (gonadal, GN), inguinal subcutaneous (SC) and *de novo* formed fat pads were removed and weighed; portions were snap-frozen in liquid nitrogen for RNA extraction and paraffin sections (7 μm) were prepared for histology.

#### Diet induced obesity

Male C57BL6/N mice, from the age of 5 weeks on, were kept in microisolation cages on a 12h day/night cycle and fed for 15 weeks with a HFD or a standard fat diet (SFD, KM-04-k12, Muracon, Carfil, Oud-Turnhout, Belgium; 13% kcal as fat, caloric value 10.9 kJ/g). At the end of the experiments SC and GN adipose tissues were collected and treated as described above.

#### Isolation of adipocytes, stromal vascular fractions and endothelial cells

*De novo* formed fat pads from NUDE mice and SC and GN adipose tissues from lean (SFD) and obese (HFD) WT (C57BL6/N) mice were obtained as described above and used to separate adipocytes from the stromal vascular cell fraction (SVF) by collagenase treatment, as described elsewhere [23, 24]. The two cell populations were used for RNA extraction.

To further isolate microvascular endothelial cells (MEC), the SVF was filtered through a 40-μm cell strainer, transferred to Histopaque-1077 solution (Sigma-Aldrich) and centrifuged at 400 *g*. To obtain pure MEC, a combination of two immunomagnetic selections was performed; first a negative selection (rat anti-mouse CD45 antibody, Biolegend; San Diego, CA) for enrichment of CD45^-^ cells containing MEC, followed by a positive selection for the purification of MEC, using specific markers including CD31 (rat anti-mouse CD31, Biolegend), CD102 (rat anti-mouse CD102 antibody, Biolegend) and isolectin B4 (FITC-labeled isolectin B4, GSI-B4, Vector Laboratories; Burlingame, CA) [25]. After separation, the freshly isolated MEC were cultured in EGM-2MV media (Lonza, Walkersville, MD) supplemented with 10% FBS (Lonza) on dishes coated with 1.5% gelatin (Sigma-Aldrich). MEC were collected for RNA extraction.

All animal experiments were approved by the local ethical committee of the University of Leuven (KU Leuven, Leuven, Belgium) (KU Leuven P082-2011) and performed in accordance with the NIH Guide for the Care and use of Laboratory Animals (1996).

### Assays

#### Gene expression studies

Isolation of total RNA from differentiated cells and SC, GN and *de novo* adipose tissue as well as isolated cell fractions was performed using the RNeasy Mini kit (Qiagen, Basel, Switzerland) according to the manufacturer’s protocol.

mRNA expression levels were determined by quantitative real time PCR, as described elsewhere [26]. The sequences of primers and probes used for *Pref1*, *GPDH*, *PPAR-γ* and *aP2* are described elsewhere [26]. Taqman gene expression assays (ThermoFisher Scientific) were used to amplify adiponectin (Mm00456425_m1), CD36 (Mm00432403_m), F4/80 (Mm00802529_m1), Notch1 (Mm004352249_m1), Notch 2 (Mm00803077_m1), Hey1 (Mm00468865_m1), Hey2 (Mm00469280_m1), Hes1 (Mm01342805_m1), CAAT enhancer binding protein alpha *(*C/EBPα; Mm00514283_s1), beta *(*C/EBPβ; Mm00843434_s1), delta (C/EBPδ; Mm00786711_s1) and the housekeeping gene ß-actin (ßact, Mm01205647_g1).

Analyses were performed by the delta-delta CT method using the 7500 System SDS software (ThermoFisher Scientific); fold changes were calculated as 2^-deltadeltaCT^ relative to control cells on day 0 for *in vitro* experiments, relative to fat pads formed by control cells for *de novo* adipogenesis models, or relative to WT mice on SFD for *in vivo* studies.

#### Histological analysis

The size and density of adipocytes or blood vessels in the adipose tissues were determined by staining with haematoxylin/eosin under standard conditions or with the *Bandeiraea Simplicifolia* lectin (Sigma-Aldrich), [22, 27, 28]. Macrophages were stained with an F4/80 antibody (Serotec, Puchheim, Germany) followed by signal amplification with the tyramide signal amplification biotin system (Perkin Elmer, Waltham, MA) and visualization through the use of a streptavidin-enzyme conjugate, followed by diaminobenzidine (DAB). Subsequently, the macrophages were quantified as stained area per total section area. Collagen content was determined by staining with Sirius red and quantified as percentage stained area per total section area [29]. Analyses were performed using a Zeiss Axioplan 2 microscope with the AxioVision rel. 4.8 software (Carl Zeiss, Oberkochen, Germany). For each animal at least 3 pictures from at least 3 sections were made.

#### COUP-TFII protein determination

Protein levels of COUP-TFII were monitored by Western bloting, under reducing conditions, using protein extracts (50 μg). Non specific binding was blocked by incubation of the membranes with 5% non fat milk at room temperature for 2 hours, followed by overnight incubation with the primary antibody against COUP-TFII (PP-H7147-00, Perseus Proteomics Inc., Tokyo, Japan) at 4°C. Goat anti-mouse IgG (1/1000; Dako, Heverlee, Belgium) conjugated with horseradish peroxidase was used as the secondary antibody and the signal was detected with a chemi-luminescence kit (ThermoFisher Scientific). ß-actin (13E5, Cell signaling Technology, Danvers, MA) was used as loading control. Blots were analysed by densitometry, using ImageJ software (http://rsbweb.nih.gov/ij/).

### Statistical analysis

Data are reported as means ± SEM. Statistical significance between groups is analyzed by non-parametric Mann-Whitney U-test or by two-way-ANOVA for time courses of expression. Correlation analysis was performed using the non-parametric Spearman rank correlation test. Values of p < 0.05 are considered statistically significant.

## Results

### Role of COUP-TFII in in vitro differentiation of preadipocytes

**Fig 1:**
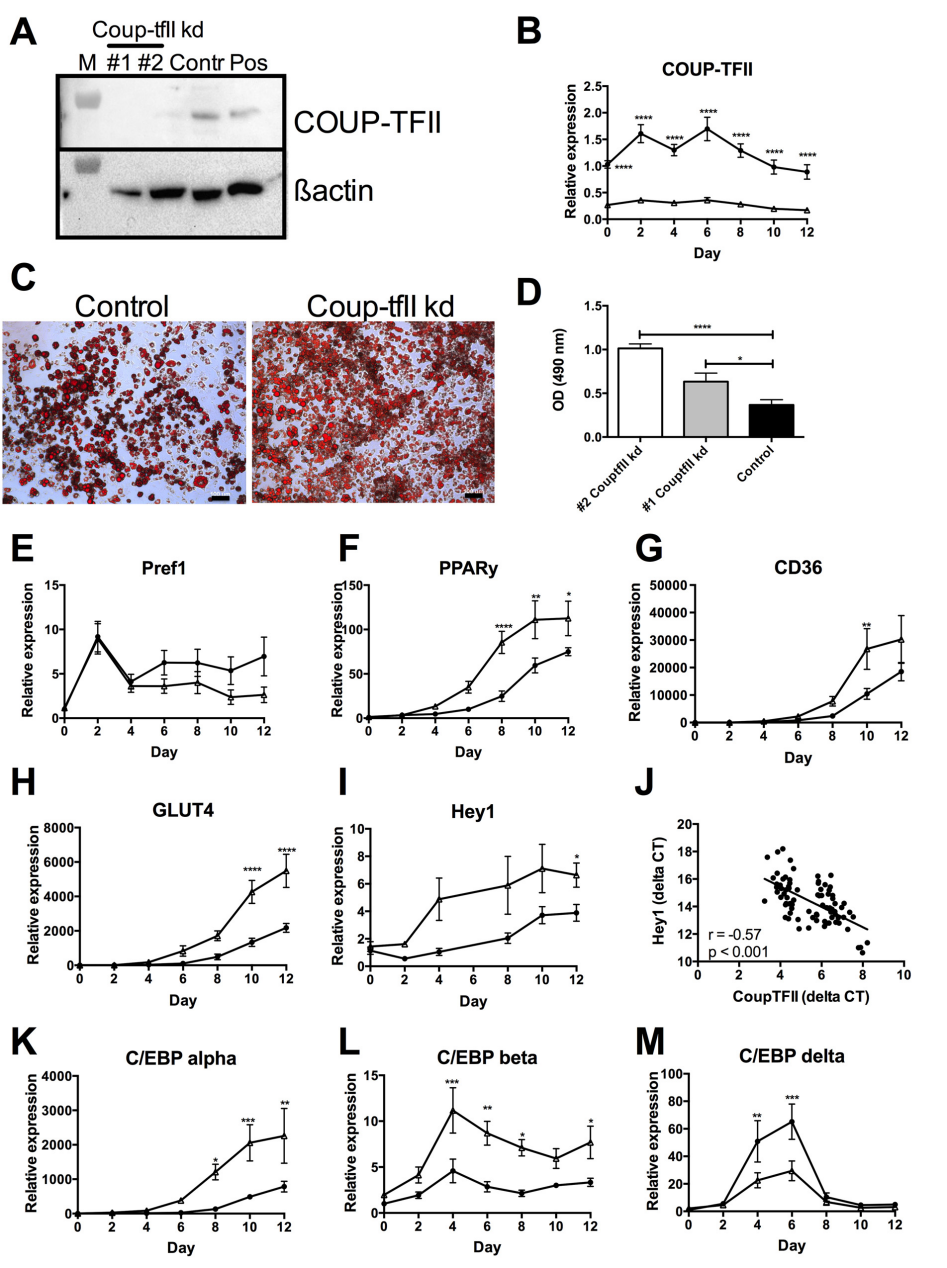
Effect of *Coup-tfII* gene silencing on *in vitro* differentiation of 3T3-F442A preadipocytes. (A) Western blotting of COUP-TFII protein in extracts of cells without (control) or with (clone #1 and *#2 CouptfII kd*) gene silencing; lane M represents the protein marker (50 kDa) and Pos represents a positive control. (B) Time course of *Coup-tfII* expression during differentiation without (⚫, black circles) or with (△, open triangles) knockdown (kd). (C-D) Oil Red O staining (C) and quantification; OD at 490 nm (D) at day 12 of differentiation. (E-H) Time course of expression of *Pref-1* (E), *PPAR-γ* (F), *CD36* (G) and *GLUT4* (H) during differentiation without (⚫, black circles) or with (△, open triangles) gene silencing. (I) Time course of *Hey1* expression without (⚫, black circles) or with (△, open triangles) *Coup-tfII* knockdown (kd). (J) Correlation between expression (delta CT levels) of *Coup-tfII* and *Hey1* with or without *Coup-tfII* knockdown. (K-M) Time course of expression of C/EBPα (K), *C/EBPβ* (L) and *C/EBPδ* (M) during differentiation without (⚫, black circles) or with (△, open triangles) gene silencing. Data are means ± SEM of 3 independent experiments; * p < 0.05; ** p<0.01; *** p<0.001; **** p<0.0001 versus control. The scale bar in panel C corresponds to 100 μm

Using Mission TRC shRNA lentiviral particles directed against *Coup-tfII*, stable knockdown of *Coup-tfII* gene expression was achieved in 3T3-F442A preadipocytes with 5 different plasmids, amounting to ≥ 70% downregulation as compared to the control plasmid SHC002V (2V) (S1A Fig). The best plasmids #*26232* (#1 CouptfII kd) and #*54475* (#2 CouptfII kd) were selected for further experiments. No significant change in *Coup-tfII* gene expression level was observed upon transduction with the control plasmid SHC002V as compared to non-transduced 3T3-F442A cells (not shown). Gene silencing was associated with a significant decrease of *Coup-tfII* (>90% for #2 CouptfII kd) at protein level in cell extracts, as confirmed by Western blotting (Fig 1A). Quantitative analysis of the ratio COUP-TFII/ ß-actin for both clones confirmed this (0.06 ± 0.04 and 0.17 ± 0.06 versus 0.75 ± 0.1, both p < 0.05 for plasmids #2 and # 1 CouptfII kd versus 2V control, respectively). *Coup-tfII* mRNA expression upon gene silencing was stable during the 12-day differentiation period (Fig 1B).

*Coup-tfII* gene silencing resulted in enhanced differentiation of 3T3-F442A preadipocytes into mature adipocytes, as visualized by Oil Red O staining and analyzed by light microscopy (Fig 1C). Quantitative analysis confirmed significantly higher intra-cytoplasmatic lipid content as compared to control (Fig 1D). Monitoring of adipogenic markers during differentiation of clone #2 CouptfII kd confirmed lower expression of *Pref-1* (Fig 1E) and enhanced expression levels of *PPAR-γ* (Fig 1F), *CD36* (Fig 1G) and *GLUT4* (Fig 1H), compatible with a higher degree of differentiation upon *Coup-tfII* gene silencing. The expression level of other pro-adipogenic transcription factors including *C/EBPα* and *C/EBPβ* was increased upon knockdown of Coup-tfII (Fig 1K and 1L), whereas *C/EBPδ* was suppresed during the early fase of differentiation (Fig 1M).

Monitoring of Notch target genes showed only higher expression of H*ey1* upon Coup-tfII gene silencing (Fig 1I), amounting to 1.7 ± 0.2 fold at day 12. A strong negative correlation was observed between H*ey1* and *Coup-tfII* expression (Fig 1J). No differences were observed regarding other Notch signaling genes including *Notch1* and *Notch2* or the target gene *Hes1* (S1B–S1D Fig).

### Role of Hey1 in in vitro differentiation of preadipocytes

To investigate the hypothesis that Hey1 is involved in the observed stimulation of adipogenesis, a stable knock-down of *Hey1* gene expression was achieved in 3T3-F442A preadipocytes, using the same approach as described above. Of the five plasmids used for transduction, the plasmid with strongest gene silencing was selected for further analysis (86482, called clone C2; S2A Fig).

**Fig 2:**
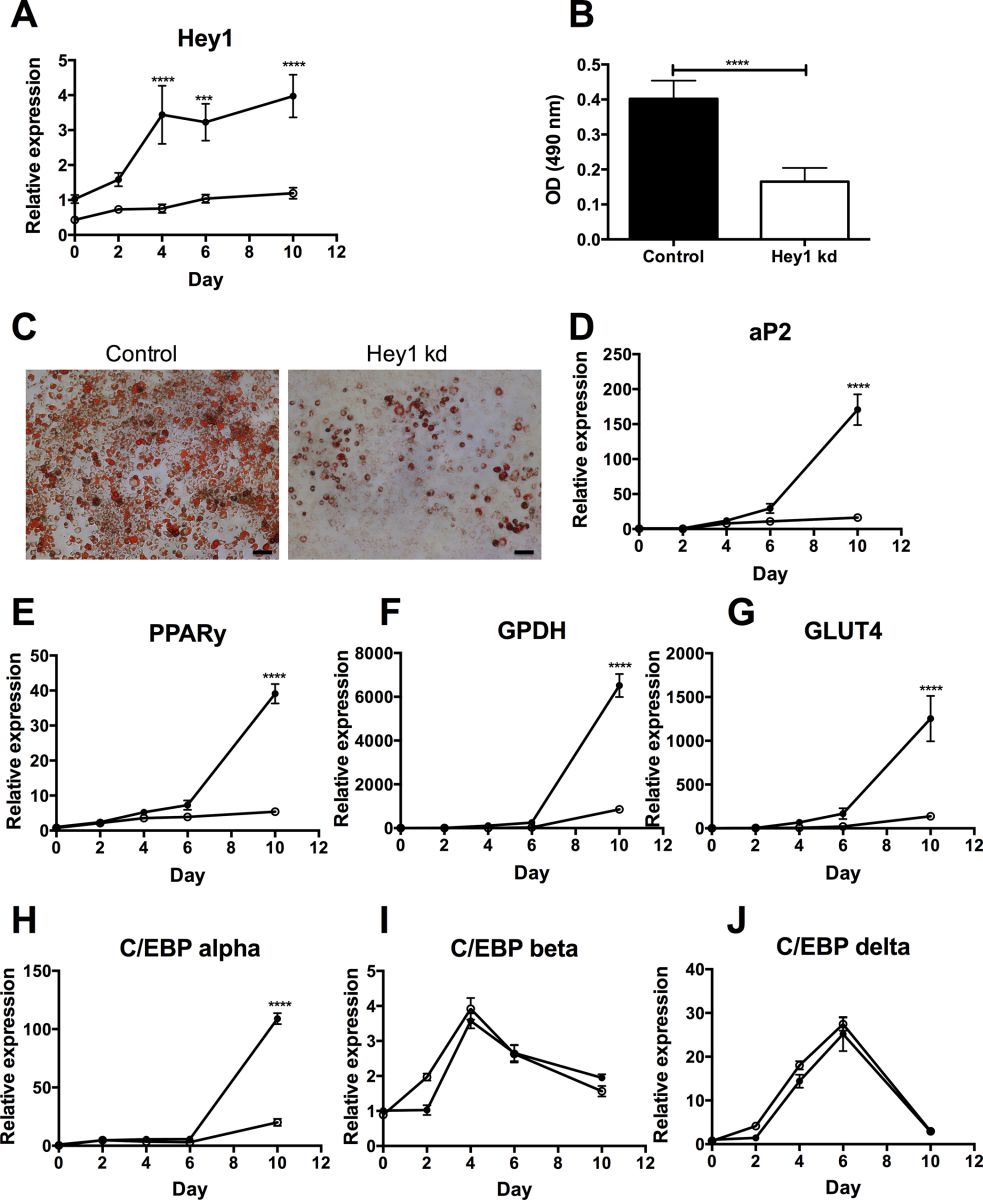
Effect of *Hey1* gene silencing on *in vitro* differentiation of 3T3-F442A preadipocytes. (A) Time course of *Hey1* expression during differentiation without (⚫, black circles) or with (○, open circles) knockdown (kd). (B-C) Oil Red O staining and quantification (OD at 490 nm) at day 12 of differentiation. (D-J) Time course of expression of *aP2* (D), *PPAR-γ* (E), *GPDH* (F), *GLUT4* (G) C/EBPα (H), *C/EBPβ* (I) and *C/EBPδ* (J) during differentiation without (⚫, black circles) or with (○, open circles) knockdown (kd). Data are means ± SEM of 3 independent experiments; *** p < 0.001 **** p < 0.0001. The scale bar in panel C corresponds to 100 μm

During differentiation of control preadipocytes, *Hey1* mRNA expression gradually increased as a function of time, whereas *Hey1* gene silencing (Fig 2A) was stable during the 12day-differentiation period. Oil Red O analysis of the intra-cytoplasmatic lipid content of differentiated preadipocytes confirmed impaired differentiation upon *Hey1* silencing (Fig 2B and 2C). Monitoring of adipogenic markers including *aP2*, *PPAR-γ*, *GPDH*, *GLUT4 and C/EBPα* during differentiation confirmed lower expression in *Hey1* knock down cells as compared to control cells (Fig 2D–2H). No differences in the expression of *C/EBPβ*, *C/EBPδ* (Fig 2I and 2J) Notch1, Notch2 or the target gene *Hes1* were detected (S2B–S2D Fig).

**Fig 3:**
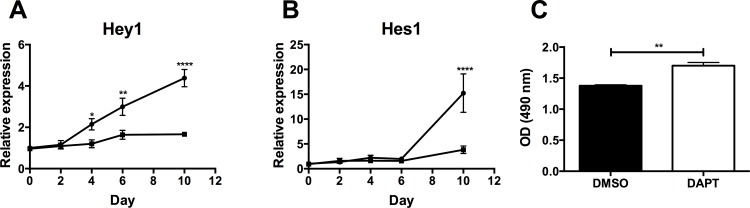
Effect of the γ-secretase inhibitor, DAPT, on differentiation of 3T3-F442A preadipocytes. (A-B) Time course of *Hey1* (A) and *Hes1* (B) during differentiation without (⚫, black circles) or with (⬛, black squares) DAPT treatment. (C) The extent of differentiation as monitored by Oil Red O staining. Data are means ± SEM of 3 independent experiments; * p < 0.05, ** p < 0.01, **** p < 0.0001.

To investigate the impact of canonical Notch signaling on adipogenesis, 3T3-F442A preadipocytes were differentiated in the presence of the γ-secretase inhibitor DAPT. Inhibitor treatment efficiently blocked Notch signaling, as *Hes1* and *Hey1* were significantly downregulated (Fig 3A and 3B), as compared to DMSO treated cells. This resulted in enhanced *in vitro* adipogenesis, as confirmed by more intra-cytoplasmatic lipid accumulation upon DAPT treatment (Fig 3C). However no marked differences were measured in relative expression levels of adipogenic markers including *aP2*, *PPAR-γ*, *GPDH*, *GLUT4* and *Pref-1* (data not shown).

Overall, these findings support our hypothesis that COUP-TFII and Hey1 play a functional role in preadipocyte differentiation, whereas canonical Notch signaling is dispensable for *in vitro* adipogenesis.

### Role of COUP-TFII in *de novo* adipogenesis *in vivo*

**Fig 4:**
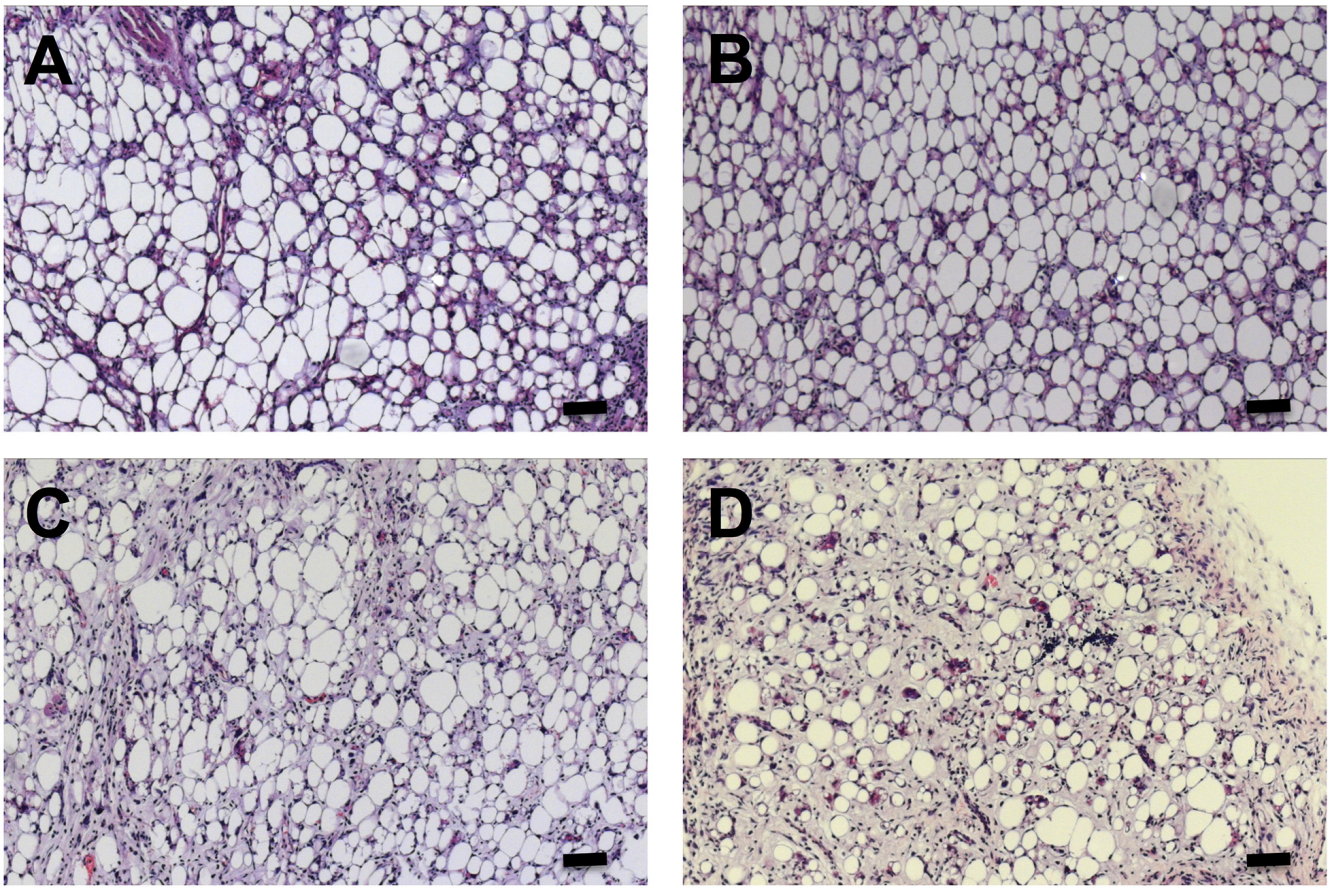
H&E Staining of *de novo* fat pads formed upon injection in NUDE mice of control preadipocytes. (A and C) or of preadipocytes with Coup-tfII knockdown (B) or Hey1 knockdown (D). The scale bar corresponds to 100 μm.

Injection of 3T3-F442A preadipocytes with (clone #2 CouptfII kd) or without (control) *Coup-tfII* gene silencing in the back of NUDE mice, resulted in the formation of *de novo* fat pads after 4 weeks of HFD feeding. Body weight gain was comparable in both groups, resulting in identical body weights and weights of isolated SC and GN fat depots (Table 1). The weight of other organs, including spleen, liver, kidney, lung, pancreas and heart was also not affected (not shown). However, the weight of the *de novo* formed fat pads from preadipocytes with *Coup-tfII* gene silencing was significantly higher as compared to controls (Table 1). Histological analysis of sections of *de novo* adipose tissue revealed no differences in the size of the adipocytes, but a larger adipocyte density in the fat pads formed from the *Coup-tfII* knockdown cells (Table 1, Fig 4A and 4B). This is consistent with more adipocytes per area, indicating more adipogenesis. Blood vessel size and density were not significantly different in both groups. Staining of adipose tissue sections with anti-F4/80 antibody indicated similar macrophage content in the sections with or without *Coup-tfII* gene silencing, and quantitative analysis confirmed comparable macrophage density. Total collagen levels, measured by Sirius Red staining, in the *de novo* formed fat pads were also similar for both groups (Table 1).

**Table 1:** Body weight and fat depots of NUDE mice injected with 3T3-F442A preadipocytes with or without COUP-TFII gene silencing and kept on HFD for 4 weeks. Data are means ± SEM of n experiments. SC, subcutaneous; GN, gonadal; COUP-TFII kd (COUP-TFII gene silencing).

	COUP-TFII kd (n = 15)	Control (n = 13)
**Body weight start (g)**	20 ± 0.20	20 ± 0.30
**Body weight end (g)**	23.9 ± 0.50	22.6 ± 0.40
**Body weight gain (g)**	4.0 ± 0.30	4.0 ± 0.20
**SC fat (mg)**	225 ± 13	188 ± 17
**GN fat (mg)**	378 ± 13	309 ± 29
**De novo fat**		
Weight (mg)	34 ± 1.6**	27 ± 1.4
Adipocyte size (μm^2^)	736 ± 56	681 ± 39
Adipocyte density (x10^-6^/μm^2^)	559 ± 50*	458 ± 41
Blood vessel size (μm^2^)	22 ± 2.0	22 ± 1.0
Blood vessel density (x10^-6^/μm^2^)	747 ± 93	649 ± 59
Macrophage content (%)	1.7 ± 0.86	1.8 ± 0.34
Collagen content (%)	35 ± 1.8	31 ± 1.0

* and ** p < 0.05 and p < 0.001 versus control (Mann-Withney U-test)

However, overall *Coup-tfII* expression in the *de novo* fat pads was similar with or without gene silencing in the preadipocytes (1.02 ± 0.07 vs 1.1 ± 0.08 for 2V vs Coup-tfII kd; p > 0.05, see below). To further confirm our hypothesis that *Coup-tfII* gene silencing enhances *in vivo* adipogenesis we monitored relative aP2 mRNA expression, showing significantly upregulated aP2 expression in the fat pads formed upon injection of Coup-tfII knockdown cells as compared to control fat pads (S3A Fig).

### Role of Hey1 in *de novo* adipogenesis *in vivo*

To further investigate the effect of Hey1 on *de novo* adipogenesis, 3T3-F442A preadipocytes with (clone C2) or without (control) *Hey1* gene silencing were injected in the back of NUDE mice, resulting in the formation of *de novo* fat pads after 4 weeks of HFD feeding. Body weights and weights of isolated SC and GN fat depots were not different between both groups. As expected, *de novo* adipogenesis was impaired upon *Hey1* gene silencing, shown by a lower fat pad mass and a smaller size of the adipocytes and lower adipocyte density (Table 2, Fig 4C and 4D). Monitoring of overall *Hey1* expression in the *de novo* fat pads showed a higher expression in the fat pads formed after injection of *Hey1* knockdown cells (1.0 ± 0.06 vs 1.3 ± 0.07 for 2V vs Hey1 kd; p = 0.02, see below). To further confirm our hypothesis that *Hey1* gene silencing inhibits *in vivo* adipogenesis we monitored relative aP2 mRNA expression, showing significantly decreased aP2 expression in the fat pads formed upon injection of Hey1 knockdown cells as compared to control fat pads (S3B Fig).

**Table 2:** Body weight and fat depots of NUDE mice injected with 3T3-F442A preadipocytes with or without Hey1 gene silencing and kept on HFD for 4 weeks. Data are means ± SEM of n experiments. SC, subcutaneous; GN, gonadal; Hey1 kd (Hey1 gene silencing).

	Hey1 kd (n = 8)	Control (n = 8)
**Body weight start (g)**	23 ± 0.75*	20 ± 0.60
**Body weight end (g)**	24 ± 0.61	24 ± 0.47
**Body weight gain (g)**	2.0 ± 0.30	3.0 ± 0.60
**SC fat (mg)**	190 ± 21	204 ± 19
**GN fat (mg)**	350 ± 40	298 ± 18
**De novo fat**		
Weight (mg)	12 ± 0.75***	21 ± 1.6
Adipocyte size (μm^2^)	404 ± 46***	879 ± 43
Adipocyte density (x10^-6^/μm^2^)	124 ± 20***	385 ± 44

* and *** p < 0.05 and p < 0.0001 versus control (Mann-Withney U-test).

Overall, these data indicate that Hey1 plays an important functional role in *in vivo* adipogenesis.

### COUP-TFII and Hey1 expression in adipose tissues

**Fig 5:**
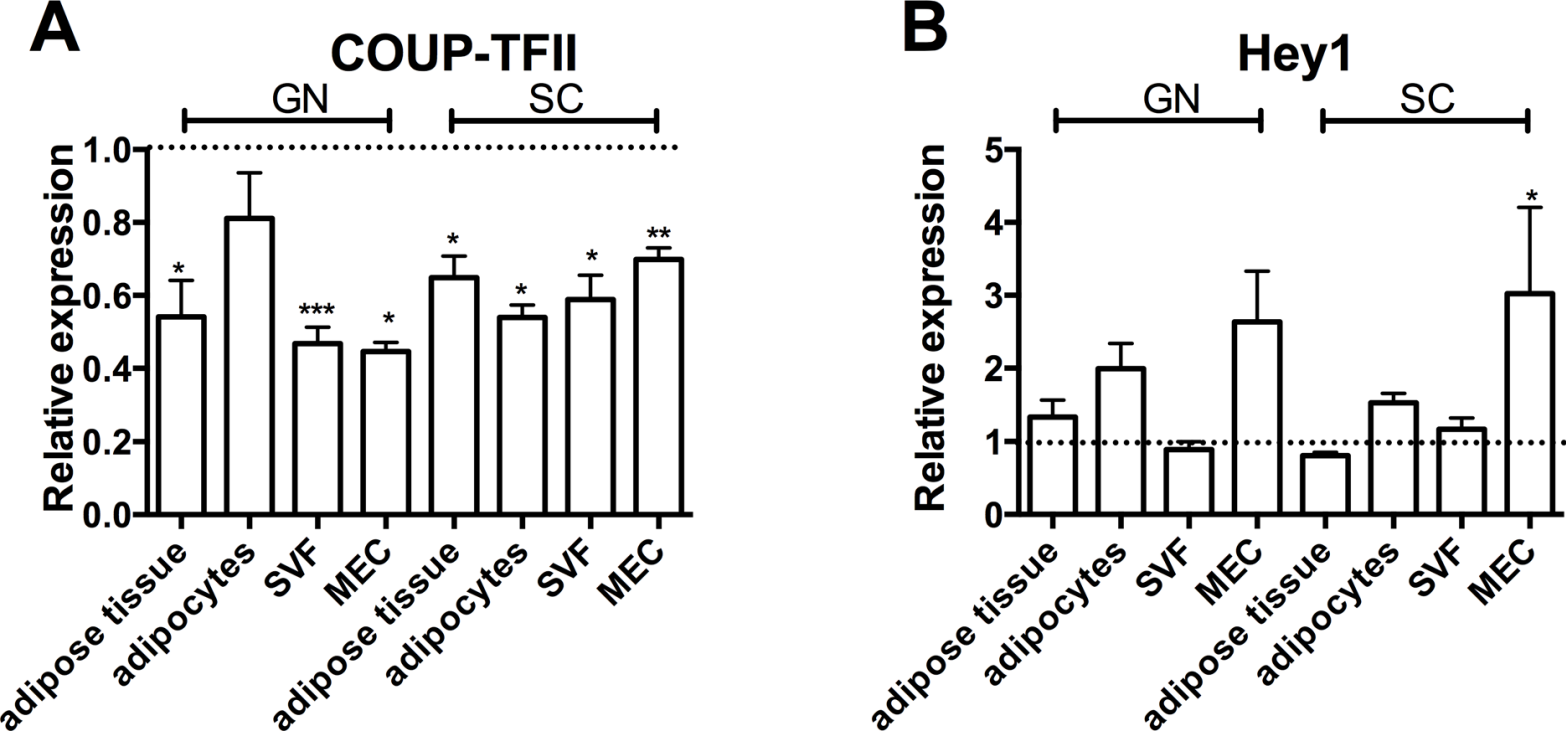
Expression of *Coup-tfII* and *Hey1* in adipose tissue and isolated cell fractions. Expression of *Coup-tfII* (A) or *Hey1* (B) in gonadal (GN) and subcutaneous (SC) adipose tissues, as well as in isolated adipocytes and stromal vascular fractions (SVF) and in microvascular endothelial cells (MEC) derived from SC and GN adipose tissues obtained from obese mice, is shown relative to samples from lean mice (dotted line). Data are means ± SEM of at least 4 samples; * p < 0.05, ** p < 0.01, *** p < 0.001.

To evaluate *Coup-tfII* and *Hey1* expression in adipose tissue and isolated adipocytes during development of obesity, male C57BL6/N mice, from the age of 5 weeks on, were kept on SFD or HFD for 15 weeks. *Coup-tfII* expression in GN and SC adipose tissue was lower upon HFD as compared to SFD feeding (Fig 5A). A strong negative correlation was observed between *Coup-tfII* expression and the GN (r = -0.81; p = 0.002) or SC (r = -0.77; p = 0.003) adipose tissue mass. However, *Hey1* expression was only slightly increased in GN fat upon HFD feeding (Fig 5B). In isolated adipocytes from SC or GN fat of obese mice, expression of *Coup-tfII* was lower as compared to those from lean mice (Fig 5A), whereas expression of *Hey1* was slightly but not significantly higher (p = 0.07 or p = 0.09 for SC or GN) (Fig 5B). In addition, relative expression of *Coup-tfII* was also decreased in the SVF from obese SC or GN adipose tissues, whereas Hey1 expression in SVF did not differ between obese and lean mice (Fig 5A and 5B). Purity of isolated adipocytes and SVF was confirmed by on the expression of the adipocyte specific marker *adiponectin* and the macrophage marker *F4/80* (data not shown).

As adipocytes and endothelial cells may have a common progenitor, we also investigated the expression of these genes in isolated MEC from SC and GN adipose tissues from lean and obese mice. Similar as in adipocytes, *Coup-tfII* expression (Fig 5A) was significantly lower, whereas *Hey1* expression (Fig 5B) was higher in isolated MEC from SC or GN adipose tissues of obese mice as compared to those of lean controls. Overall, *Coup-tfII* and *Hey1* appear to follow a similar expression pattern in adipose tissue, adipocytes and endothelial cells upon induction of diet-induced obesity.

In addition, we monitored expression of *Coup-tfII* and *Hey1* in isolated adipocytes and SVF of *de novo* fat pads formed following injection of 3T3-F442A preadipocytes in the back of NUDE mice. Purity of the cell fractions was evidenced by higher expression of the adipocyte specific adipokine, *adiponectin* (230 fold, p = 0.002), and the lower expression of the macrophage maker *F4/80* (3 fold, p = 0.002) in the adipocyte fraction as compared to the SVF. Expression of *Coup-tfII* (15 fold, p = 0.002) as well as *Hey1* (8 fold, p = 0.002) was significantly higher in the SVF than in the adipocyte fraction, indicating that these genes are expressed by other cells in the *de novo* fat pads besides adipocytes. This probably explains our observation that overall *Coup-tfII* and *Hey1* expression in *de novo* fat pads originating from 3T3-F442A preadipocytes with gene silencing was not significantly downregulated as compared to controls (see above).

## Discussion

COUP-TFII belongs to the steroid/thyroid hormone receptor superfamily and may contribute to the pathogenesis of obesity. It has not conclusively been established, however, whether its role is pro- or anti-adipogenic. Indeed, several groups reported an anti-adipogenic role of COUP-TFII in adipogenesis based on *in vitro* experiments [4, 5]. This is in contrast with the only *in vivo* data available showing that heterozygous COUP-TFII mice have less adipose tissue as compared to wild-type mice [2]. As far as we know, we are the first group to investigate the role of COUP-TFII in adipogenesis based on a combination of established mouse models of *in vitro* adipocyte differentiation and *in vivo* adipogenesis. In agreement with the results of Okamura et al. [4] and Xu et al. [5], we found that gene silencing of *Coup-tfII* in 3T3-F442A preadipocytes, resulted in enhanced differentiation into mature adipocytes. I*n vivo*, *de novo* fat pad formation in NUDE mice was significantly stimulated following injection of preadipocytes with *Coup-tfII* gene silencing. Interestingly, in our study, *Coup-tfII* gene silencing was associated with up-regulation of the Notch signaling target gene *Hey1*. A functional role of Hey1 in adipogenesis was further confirmed by gene silencing in 3T3-F442A preadipocytes, resulting in impaired differentiation and in reduced *de novo* adipogenesis. Interestingly, this established *de novo* model combines the features of *in vitro* cell lines with the stringency of an *in vivo* environment. Furthermore, this model was previously shown to be reprsentative for *de novo* adipogenesis and adipose tissue formation [20–22]. Indeed, Mandrup et al., showed that implanted preadipocytes harboring a beta-galactosidase transgene gave rise to fat pads in which almost all adipocytes expressed beta-galactosidase [20].

It is assumed that COUP-TFII is a major regulator of Notch signaling pathways, but, conflicting data have been reported on the role of Notch signaling in adipogenesis [9–15]. Thus, Huang et al. [13] reported that blocking canonical Notch signaling enhances adipogenesis of adipose derived stem cells (ASC), an earlier stage than 3T3-F442A preadipocytes. However, Jagged-1-mediated activation of Notch signaling was also found to induce adipogenesis of ASC [12]. In contrast, Jung et al. showed that silk peptides, that lower body fat, block adipocyte differentiation (in C3H10T1/2 and 3T3-L1 cells) to a similar extent as known Notch signaling inhibitors, including DAPT [11]. DAPT is an inhibitor of canonical Notch signaling, that blocks the γ-secretase, which cleaves the Notch receptors to generate the Notch intracellular domain (NICD) that activates the transcription of several target genes. Our data with DAPT treatment show that canonical Notch signaling is dispensable for adipogenesis, indicating that repression of its target genes *Hey1*, *Hes1* and potentially others does not affect adipocyte differentiation, whereas specific gene silencing of *Hey1* does inhibit adipogenesis. Since Hey1 and Hes1 are not the only targets of Notch signaling, DAPT may affect other target genes and thereby counteract the inhibiting effect of blocking Hey1 on adipogenesis.

*Hey1* expression is increased when *Coup-tfII* is knocked down in the adipocytes, but wether this effect is due to a direct regulation of *Hey1* expression by *Coup-tfII* needs further investigation. Aranguren et al. showed that COUP-TFII homodimers have the potential to bind directly to the promoter regions of the Notch target genes *Hey1* and *Hey2* in vascular and lymphatic endothelial cells, causing transcriptional repression [7]. In addition, it was earlier stated in the literature that white adipocytes are derived from endothelial and haematopoietic lineages [30–32]. These data strengthen our findings that COUP-TFII plays an anti-adipogenic role via downregulating the Notch signaling target gene *Hey1*. To confirm this hypothesis we investigated the expression of *Coup-tfII* and *Hey1* in a model of diet induced obesity. We found that in WT mice, expression of *Coup-tfII* in adipose tissue decreased with nutritionally induced obesity and was negatively correlated with adipose tissue mass. In isolated adipocytes of obese adipose tissues, as compared to lean controls, expression of *Coup-tfII* decreased whereas that of *Hey1* increased. It was previously shown that endothelial cells and adipocytes have a common progenitor [8]. Interestingly, we found that *Coup-tfII* and *Hey1* follow a similar expression pattern in adipose tissue adipocytes and endothelial cells upon induction of diet-induced obesity.

Thus, our data are compatible with the following mechanism: COUP-TFII binds directly to the promoter of the Notch signaling target gene *Hey1* thereby blocking its promoting effect on adipocyte differentiation and adipogenesis. However, the requirement and role of the COUP-TFII/Hey1 signaling in adipose tissue development and adipocyte differentiation and its potential as future strategy to treat obesity and its related metabolic diseases need further investigation.

## Supporting Information

S1 Fig Effect of *Coup-tfII* gene silencing on *in vitro* differentiation of 3T3-F442A preadipocytes. (A) To obtain long term stable gene silencing of *Coup-tfII* in 3T3-F442A preadipocytes, five different clones were tested (TRCN0000026167-026232-054474-054475-312204). (B-D) Time course of expression of *Hes1* (B), *Notch1* (C) and *Notch2* (D) during differentiation without (⚫, black circles) or with (△, open triangles) gene silencing. Data are means SEM of 3 independent experiments; ** p<0.01 versus control 2V.
(TIFF)

S2 Fig Effect of *Hey1* gene silencing on *in vitro* differentiation of 3T3-F442A preadipocytes. (A) To obtain long term, stable gene silencing of *Hey1* in 3T3-F442A preadipocytes, five different clones were tested (TRCN0000086479-86480-86481-86482 and TRCN0000311840). (B-D) Time course of expression of *Hes1* (B), *Notch1* (C) and *Notch2* (D) during differentiation without (⚫, black circles) or with (○, open circles) gene silencing. Data are means SEM of 3 independent experiments; ** p<0.01 versus control 2V.
(TIFF)

S3 Fig Effect of *Coup-tfII* and *Hey1* gene silencing on *in vivo* adipogenesis. Expression of aP2 in *de novo* formed fat pads upon injection of 3T3-F442A preadipocytes with Coup-tfII (A) or Hey1 (B) gene silencing (kd) as compared to control fat pads (injected with 2V control 3T3-F442A preadipocytes). Data are means SEM of at least 4 samples; ** p < 0.01, **** p < 0.0001
(TIFF)

## References

[1] Flegal KM, Graubard BI, Williamson DF, Gail MH. Cause-specific excess deaths associated with underweight, overweight, and obesity. JAMA. 2007;298(17):2028–37. 17986696 10.1001/jama.298.17.2028

[2] Li L, Xie X, Qin J, Jeha GS, Saha PK, Yan J, et al The nuclear orphan receptor COUP-TFII plays an essential role in adipogenesis, glucose homeostasis, and energy metabolism. Cell Metab. 2009;9(1):77–87. 10.1016/j.cmet.2008.12.002 19117548 PMC2630393

[3] Qin J, Chen X, Xie X, Tsai MJ, Tsai SY. COUP-TFII regulates tumor growth and metastasis by modulating tumor angiogenesis. Proc Natl Acad Sci U S A. 2010;107(8):3687–92. 10.1073/pnas.0914619107 20133706 PMC2840495

[4] Okamura M, Kudo H, Wakabayashi K, Tanaka T, Nonaka A, Uchida A, et al COUP-TFII acts downstream of Wnt/beta-catenin signal to silence PPARgamma gene expression and repress adipogenesis. Proc Natl Acad Sci U S A. 2009;106(14):5819–24. 10.1073/pnas.0901676106 19307559 PMC2667001

[5] Xu Z, Yu S, Hsu CH, Eguchi J, Rosen ED. The orphan nuclear receptor chicken ovalbumin upstream promoter-transcription factor II is a critical regulator of adipogenesis. Proc Natl Acad Sci U S A. 2008;105(7):2421–6. 10.1073/pnas.0707082105 18250317 PMC2268152

[6] Chen X, Qin J, Cheng CM, Tsai MJ, Tsai SY. COUP-TFII is a major regulator of cell cycle and Notch signaling pathways. Mol Endocrinol. 2012;26(8):1268–77. 10.1210/me.2011-1305 22734039 PMC3404301

[7] Aranguren XL, Beerens M, Coppiello G, Wiese C, Vandersmissen I, Lo Nigro A, et al COUP-TFII orchestrates venous and lymphatic endothelial identity by homo- or hetero-dimerisation with PROX1. J Cell Sci. 2013;126(Pt 5):1164–75. 10.1242/jcs.116293 23345397

[8] Planat-Benard V, Silvestre JS, Cousin B, Andre M, Nibbelink M, Tamarat R, et al Plasticity of human adipose lineage cells toward endothelial cells: physiological and therapeutic perspectives. Circulation. 2004;109(5):656–63. 14734516 10.1161/01.CIR.0000114522.38265.61

[9] Garces C, Ruiz-Hidalgo MJ, Font de Mora J, Park C, Miele L, Goldstein J, et al Notch-1 controls the expression of fatty acid-activated transcription factors and is required for adipogenesis. J Biol Chem. 1997;272(47):29729–34. 9368042 10.1074/jbc.272.47.29729

[10] Nichols AM, Pan Y, Herreman A, Hadland BK, De Strooper B, Kopan R, et al Notch pathway is dispensable for adipocyte specification. Genesis. 2004;40(1):40–4. 15354292 10.1002/gene.20061

[11] Jung SR, Song NJ, Hwang HS, An JJ, Cho YJ, Kweon HY, et al Silk peptides inhibit adipocyte differentiation through modulation of the Notch pathway in C3H10T1/2 cells. Nutr Res. 2011;31(9):723–30. 10.1016/j.nutres.2011.08.010 22024497

[12] Ba K, Yang X, Wu L, Wei X, Fu N, Fu Y, et al Jagged-1-mediated activation of notch signalling induces adipogenesis of adipose-derived stem cells. Cell Prolif. 2012;45(6):538–44. 10.1111/j.1365-2184.2012.00850.x 23046039 PMC6496181

[13] Huang Y, Yang X, Wu Y, Jing W, Cai X, Tang W, et al gamma-secretase inhibitor induces adipogenesis of adipose-derived stem cells by regulation of Notch and PPAR-gamma. Cell Prolif. 2010;43(2):147–56. 10.1111/j.1365-2184.2009.00661.x 20447060 PMC6496520

[14] Ross DA, Hannenhalli S, Tobias JW, Cooch N, Shiekhattar R, Kadesch T. Functional analysis of Hes-1 in preadipocytes. Mol Endocrinol. 2006;20(3):698–705. 16282371 10.1210/me.2005-0325

[15] Ross DA, Rao PK, Kadesch T. Dual roles for the Notch target gene Hes-1 in the differentiation of 3T3-L1 preadipocytes. Mol Cell Biol. 2004;24(8):3505–13. 15060169 10.1128/MCB.24.8.3505-3513.2004 PMC381674

[16] Green H, Kehinde O. Spontaneous heritable changes leading to increased adipose conversion in 3T3 cells. Cell. 1976;7(1):105–13. 949738 10.1016/0092-8674(76)90260-9

[17] Christiaens V, Van Hul M, Lijnen HR, Scroyen I. CD36 promotes adipocyte differentiation and adipogenesis. Biochim Biophys Acta. 2012;1820(7):949–56. 10.1016/j.bbagen.2012.04.001 22507268

[18] Scroyen I, Christiaens V, Lijnen HR. No functional role of plasminogen activator inhibitor-1 in murine adipogenesis or adipocyte differentiation. J Thromb Haemost. 2007;5(1):139–45. 17067365 10.1111/j.1538-7836.2006.02284.x

[19] Ramirez-Zacarias JL, Castro-Munozledo F, Kuri-Harcuch W. Quantitation of adipose conversion and triglycerides by staining intracytoplasmic lipids with Oil red O. Histochemistry. 1992;97(6):493–7. 1385366 10.1007/BF00316069

[20] Mandrup S, Loftus TM, MacDougald OA, Kuhajda FP, Lane MD. Obese gene expression at in vivo levels by fat pads derived from s.c. implanted 3T3-F442A preadipocytes. Proc Natl Acad Sci U S A. 1997;94(9):4300–5. 9113984 10.1073/pnas.94.9.4300 PMC20717

[21] Neels JG, Thinnes T, Loskutoff DJ. Angiogenesis in an in vivo model of adipose tissue development. FASEB J. 2004;18(9):983–5. 15084517 10.1096/fj.03-1101fje

[22] Scroyen I, Jacobs F, Cosemans L, De Geest B, Lijnen HR. Blood vessel density in de novo formed adipose tissue is decreased upon overexpression of TIMP-1. Obesity (Silver Spring). 2010;18(3):638–40. 19730423 10.1038/oby.2009.279

[23] Rodbell M. Metabolism of Isolated Fat Cells. I. Effects of Hormones on Glucose Metabolism and Lipolysis. J Biol Chem. 1964;239:375–80. 14169133

[24] Voros G, Maquoi E, Demeulemeester D, Clerx N, Collen D, Lijnen HR. Modulation of angiogenesis during adipose tissue development in murine models of obesity. Endocrinology. 2005;146(10):4545–54. 16020476 10.1210/en.2005-0532

[25] Kajimoto K, Hossen MN, Hida K, Ohga N, Akita H, Hyodo M, et al Isolation and culture of microvascular endothelial cells from murine inguinal and epididymal adipose tissues. J Immunol Methods. 2010;357(1–2):43–50. 10.1016/j.jim.2010.03.011 20307543

[26] Scroyen I, Cosemans L, Lijnen HR. Effect of tissue inhibitor of matrix metalloproteinases-1 on in vitro and in vivo adipocyte differentiation. Thromb Res. 2009;124(5):578–83. 10.1016/j.thromres.2009.06.020 19608218

[27] Laitinen L. Griffonia simplicifolia lectins bind specifically to endothelial cells and some epithelial cells in mouse tissues. Histochem J. 1987;19(4):225–34. 3597137 10.1007/BF01680633

[28] Van Hul M, Lijnen HR. A functional role of gelatinase A in the development of nutritionally induced obesity in mice. J Thromb Haemost. 2008;6(7):1198–206. 10.1111/j.1538-7836.2008.02988.x 18433461

[29] Demeulemeester D, Scroyen I, Voros G, Snoeys J, De Geest B, Collen D, et al Overexpression of tissue inhibitor of matrix metalloproteinases-1 (TIMP-1) in mice does not affect adipogenesis or adipose tissue development. Thromb Haemost. 2006;95(6):1019–24. 16732382 10.1160/TH05-11-0742

[30] Crossno JT Jr., Majka SM, Grazia T, Gill RG, Klemm DJ. Rosiglitazone promotes development of a novel adipocyte population from bone marrow-derived circulating progenitor cells. J Clin Invest. 2006;116(12):3220–8. 17143331 10.1172/JCI28510 PMC1679707

[31] Sera Y, LaRue AC, Moussa O, Mehrotra M, Duncan JD, Williams CR, et al Hematopoietic stem cell origin of adipocytes. Exp Hematol. 2009;37(9):1108–20, 20 e1-4. 10.1016/j.exphem.2009.06.008 19576951 PMC2740899

[32] Tran KV, Gealekman O, Frontini A, Zingaretti MC, Morroni M, Giordano A, et al The vascular endothelium of the adipose tissue gives rise to both white and brown fat cells. Cell Metab. 2012;15(2):222–9. 10.1016/j.cmet.2012.01.008 22326223 PMC3278718

