# Supplementary figures and images for: The Anti-Adipogenic Potential of COUP-TFII Is Mediated by Downregulation of the Notch Target Gene Hey1
Source: PLoS One. 2015 Dec 31;10(12):e0145608. doi: 10.1371/journal.pone.0145608 (PMC4697848; doi:10.1371/journal.pone.0145608)

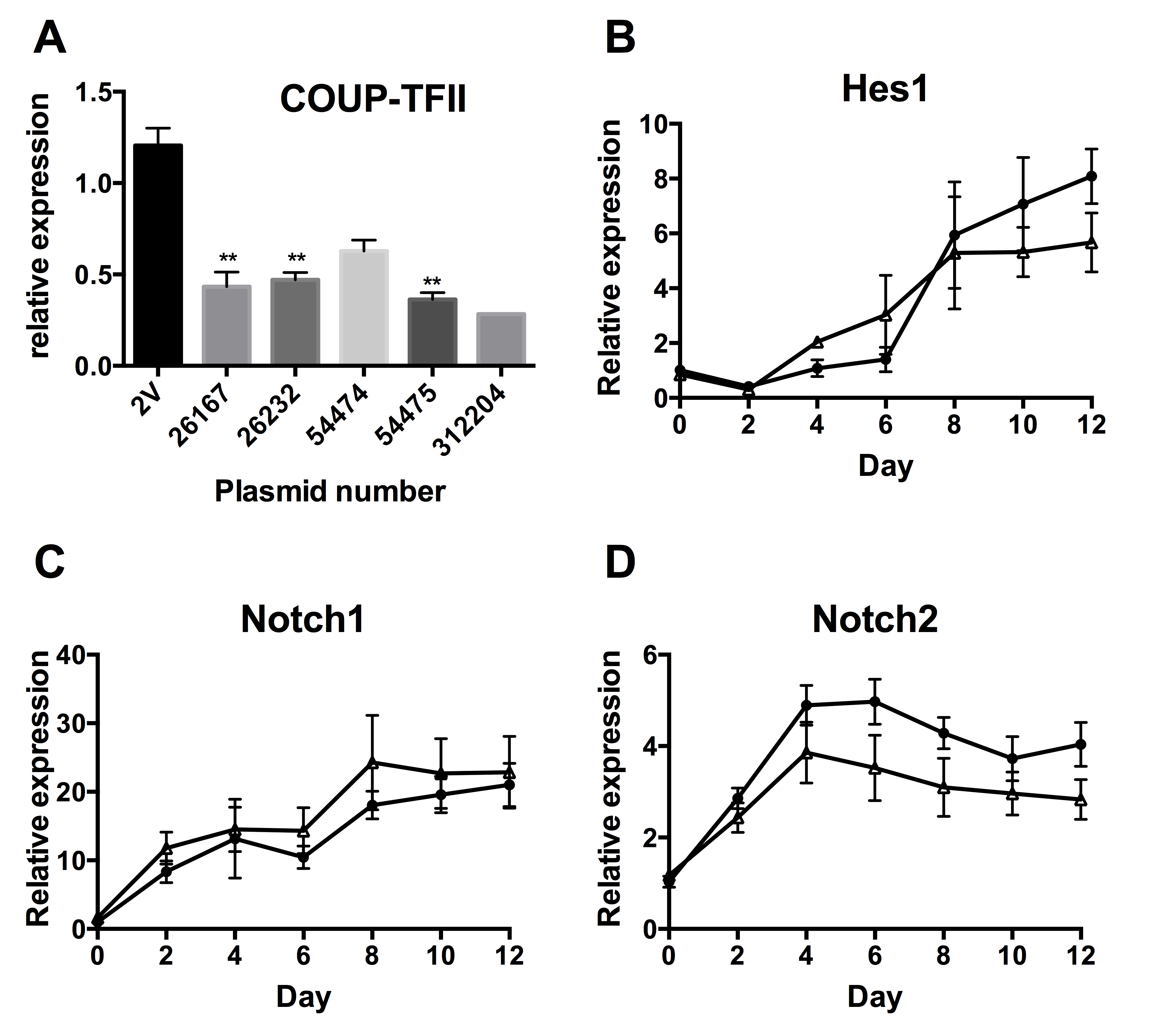

Supplement: S1 Fig — (A) To obtain long term stable gene silencing of Coup-tfII in 3T3-F442A preadipocytes, five different clones were tested (TRCN0000026167-026232-054474-054475-312204). (B-D) Time course of expression of Hes1 (B), Notch1 (C) and Notch2 (D) during differentiation without (⚫, black circles) or with (△, open triangles) gene silencing. Data are means SEM of 3 independent experiments; ** p<0.01 versus control 2V. (TIFF) [file pone.0145608.s001.tiff]

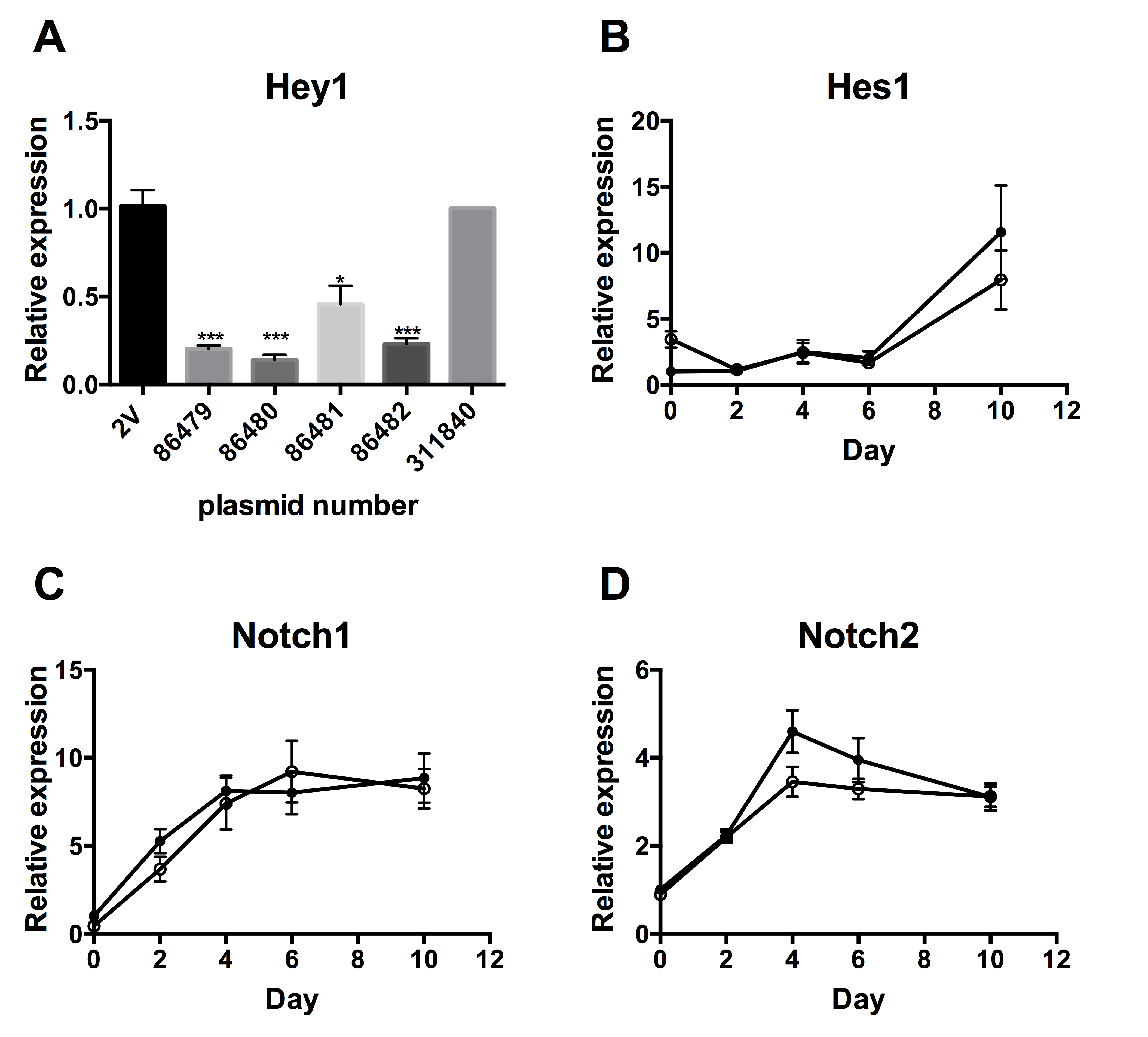

Supplement: S2 Fig — (A) To obtain long term, stable gene silencing of Hey1 in 3T3-F442A preadipocytes, five different clones were tested (TRCN0000086479-86480-86481-86482 and TRCN0000311840). (B-D) Time course of expression of Hes1 (B), Notch1 (C) and Notch2 (D) during differentiation without (⚫, black circles) or with (○, open circles) gene silencing. Data are means SEM of 3 independent experiments; ** p<0.01 versus control 2V. (TIFF) [file pone.0145608.s002.tiff]

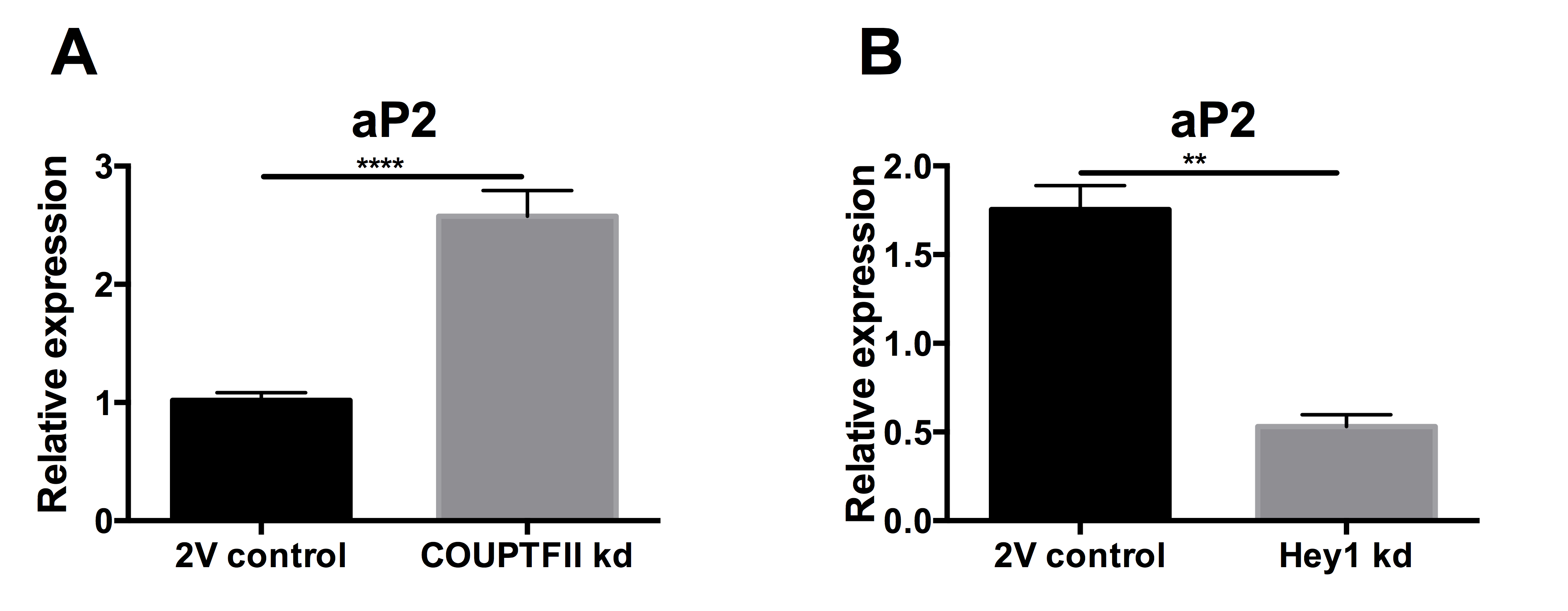

Supplement: S3 Fig — Expression of aP2 in de novo formed fat pads upon injection of 3T3-F442A preadipocytes with Coup-tfII (A) or Hey1 (B) gene silencing (kd) as compared to control fat pads (injected with 2V control 3T3-F442A preadipocytes). Data are means SEM of at least 4 samples; ** p < 0.01, **** p < 0.0001 (TIFF) [file pone.0145608.s003.tiff]
